# Checklist of the ‘lower Brachycera’ of Finland: Tabanomorpha, Asilomorpha and associated families (Diptera)

**DOI:** 10.3897/zookeys.441.7198

**Published:** 2014-09-19

**Authors:** Jere Kahanpää, Kaj Winqvist, Theo Zeegers

**Affiliations:** 1 Finnish Museum of Natural History, Zoology Unit, P.O. Box 17, FI–00014 University of Helsinki, Finland; 2 Mikonkatu 3 C 52, FI–20100 Turku, Finland; 3 Eikenlaan 24, 3768 EV Soest, the Netherlands

**Keywords:** Species list, Finland, Diptera, biodiversity, faunistics

## Abstract

A checklist of the ‘lower Brachycera’ of Finland is presented. This part of the complete checklist of Finnish Diptera covers the families Acroceridae, Asilidae, Athericidae, Bombyliidae, Mythicomyiidae, Rhagionidae, Scenopinidae, Stratiomyidae, Tabanidae, Therevidae, Xylomyidae and Xylophagidae.

## Introduction

This part of the checklist of the Diptera of Finland covers non-eremoneuran true flies (Diptera: Brachycera). The brachyceran flies excluded from the clade Eremoneura are often called the ‘lower Brachycera’ due to their basal position in the true fly tree of life. It remains unclear whether this assemblage of families is a monophyletic clade. There are also several models for the relative relationships of the various superfamilies and families. A simple classification scheme following Marshall (2012) is adopted for this checklist. Only two infraorders, Tabanomorpha and Asilomorpha, are recognized. The presentation order of families follows Woodley et al. (2009).

World catalogues have recently been published for Stratiomyidae (Woodley 2001, 2011b), Xylomyidae (Woodley 2011a), Xylophagidae (Woodley 2011c), Bombyliidae (Evenhuis and Greathead 1999, 2003) and Mythicomyiidae (Evenhuis 2002). The Finnish species were last listed by Kahanpää and Winqvist (2005). Five species have been added since the last checklist: *Haematopota italica* Meigen, 1804, *Lasiopogon septentrionalis* Lehr, 1984, *Nemotelus infortunatus* Kahanpää, 2010, *Xylophagus inermis* Krivosheina & Krivosheina, 2000 and *Zabrachia tenella* (Jaennicke, 1866) (see Kahanpää 2013, Cannings and Kahanpää 2013, Kahanpää 2010, this paper, and Krivosheina and Rozkošný 1990 respectively). Table 1 summarizes the current family species counts for the world, Europe (based on Fauna Europaea), and Finland.

**Table 1.** Number of species in tabanomorph and asilomorph families plus Acroceridae.

Family	Number of species in			Level of knowledge
	World	Europe	Finland	
*Tabanomorpha*:				
Stratiomyidae	2715 (Woodley 2001, 2011b)	141	29	good
Xylomyidae	138 (Woodley 2011a)	13–14	1	good
Xylophagidae	134 (Woodley 2011c)	8	5	average–good
Rhagionidae	694 (Pape et al. 2011)	85	15–16	average–good
Athericidae	124 (Pape et al. 2011)	10	1	good
Tabanidae	4405 (Pape et al. 2011)	213	38–39	good
*Asilomorpha*:				
Asilidae	7513 (Pape et al. 2011)	524	35	good
Bombyliidae	~5000 (Evenhuis and Greathead 1999, 2003)	335	18–19	good
Mythicomyiidae	~330 (Evenhuis 2002, Pape et al. 2011)	30	1	average
Scenopinidae	416 (Pape et al. 2011)	17	3	good
Therevidae	1129 (Pape et al. 2011)	99	17	average–good
*unplaced*:				
Acroceridae	392 (Pape et al. 2011)	34	5	average

### Tabanomorpha

The stratiomyoid and xylophagoid lineages are often treated as infraorders (Woodley et al. 2009).

The soldierflies (Stratiomyidae) are very diverse in the tropics but the species diversity decreases sharply towards the higher latitudes. The wood soldier flies (xylomyids) is a small fly family associated with dead wood. The Fauna Entomologica Scandinavica series has a volume on stratiomyoid flies (Rozkošný 1973). A new *Nemotelus* species was recently described from Finland (Kahanpää 2010a).

The Finnish rhagionids are relatively well known but a few additional species could occur in the country. *Ptiolina* is a problematic genus and the number of recognized species in Northern Europe has varied from two to seven during the last century. Athericidae was traditionally placed as a subfamily of Rhagionidae, but it seems more closely associated with Tabanidae (Marshall 2012). Itämies et al. (1990, 1993) have studied the distribution of *Atherix ibis* in Finland.

The Finnish xylophagid fauna is relatively well sampled. Adults of the North European species can be identified using Nartshuk (1988) or Kahanpää (2009). The larvae can also be identified at least at the last larval stage (Stubbs and Drake 2001, Krivosheina and Krivosheina 1966).

The tabanid nomenclature (especially *Hybomitra*) is quite convoluted and records in older publications must be taken with a grain of salt. Karvonen (1969) summarized the distribution of tabanids in Finland, but this work is now partially obsolete due to the difficulties in identifying *Hybomitra* and *Haematopota* before Chvála et al. (1972) was published. For identification of North European tabanids Chvála et al. (1972) complemented with pictures in Zeegers and van Haaren (2000) or Krčmar et al. (2011) is recommended. An illustrated guide to the Finnish species is in preparation (A. Haarto, unpublished).

### Asilomorpha

The asilids and bombyliids of Finland are rather well known from a faunistic point of view but little is known about their ecology. Most of the North European species are easy to identify but problems with *Villa* resulted in a cascade of name changes in the late 20th century. Falck (2009) and Blöchlinger (2008) are good starting points for identifying *Villa* adults. François (1969) has male genitalia figures for some of the more difficult *Villa* species. The Mythicomyiidae or micro bee flies were long seen as a subfamily of Bombyliidae.

Identifying *Thereva* species was also fraught with difficulties in the past but by the end of the 20th century the North European fauna was pretty well understood. A review of the Finnish therevid fauna with keys has recently been published (Haarto and Winqvist 2006). The window flies, Scenopinidae, is a smallish asiloid lineage associated with the therevids. It has even been proposed they are a specialized subgroup of the Therevidae (Woodley 2009).

### Acroceridae

The small-headed flies (Acroceridae) are a fly family of obscure origin. Affinities with Nemestrinidae, Tabanoidea, Stratiomyoidea, Bombyliidae and Asilomorpha have been proposed (see Marshall 2012 for further discussion). Finnish acrocerid records are mostly of single adults caught by sweep-netting, although Storå (1956) found groups of 20–40 *Acrocera orbiculus* swarming on a coastal meadow. The acrocerid species seem to have declined in abundance during the 20th century. Four of our five Finnish species are now on the national red list (Kahanpää 2010b).

## Checklist part 1: Tabanomorpha (*sensu lato*)

suborder Brachycera Macquart, 1834

clade Orthorrapha Brauer, 1863

superfamily Stratiomyoidea Latreille, 1802

**STRATIOMYIDAE** Latreille, 1802

BERIDINAE Westwood, 1838

***BERIS*** Latreille, 1802

*Beris chalybata* (Forster, 1771)

*Beris clavipes* (Linnaeus, 1767)

*Beris fuscipes* Meigen, 1820

*Beris hauseri* Stuke, 2004

= *strobli* auct. nec. Dušek & Rozkošný, 1968

*Beris morrisii* Dale, 1841

NEMOTELINAE Kertész, 1912

***NEMOTELUS*** Geoffroy, 1762

**sg. *Camptopelta*** Williston, 1917

*Nemotelus nigrinus* Fallén, 1817

**sg. *Nemotelus*** Geoffroy, 1762

*Nemotelus infortunatus* Kahanpää, 2010

*Nemotelus notatus* Zetterstedt, 1842

*Nemotelus uliginosus* (Linnaeus, 1767)

PACHYGASTRINAE Loew, 1856

***BERKSHIRIA*** Johnson, 1914

= ***Pseudowallacea*** Kertész, 1921

*Berkshiria hungarica* (Kertesz, 1921)

= *albistylum* misid.

= *barovskii* misid.

***NEOPACHYGASTER*** Austen, 1901

*Neopachygaster meromelas* (Dufour, 1841)

= *orbitalis* (Wahlberg, 1854)

***ZABRACHIA*** Coquillett, 1901

*Zabrachia minutissima* (Zetterstedt, 1838)

*Zabrachia tenella* (Jaennicke, 1866) see Notes

SARGINAE Walker, 1834

***CHLOROMYIA*** Duncan, 1837

*Chloromyia formosa* (Scopoli, 1763)

***MICROCHRYSA*** Loew, 1855

*Microchrysa cyaneiventris* (Zetterstedt, 1842)

*Microchrysa flavicornis* (Meigen, 1822)

*Microchrysa polita* (Linnaeus, 1758)

***SARGUS*** Fabricius, 1798

*Sargus cuprarius* (Linnaeus, 1758)

*Sargus flavipes* Meigen, 1822

= *nigripes* Zetterstedt, 1842

= *splendens* auct. nec. Meigen, 1804

*Sargus iridatus* (Scopoli, 1763)

*Sargus rufipes* Wahlberg, 1854

STRATIOMYINAE Latreille, 1802

tribe Oxycerini Enderlein, 1914

***OXYCERA*** Meigen, 1803

*Oxycera centralis* Loew, 1863

= *centralis* Frey, 1911 preocc.

= *freyi* Lindner, 1938

*Oxycera dives* Loew, 1845

*Oxycera trilineata* (Linnaeus, 1767)

tribe Stratiomyini Latreille, 1802

***ODONTOMYIA*** Meigen, 1803

*Odontomyia angulata* (Panzer, 1798)

*Odontomyia argentata* (Fabricius, 1794)

*Odontomyia microleon* (Linnaeus, 1758)

***OPLODONTHA*** Rondani, 1863

*Oplodontha viridula* (Fabricius, 1775)

***STRATIOMYS*** Geoffroy, 1762

*Stratiomys singularior* (Harris, 1776)

= *furcata* Fabricius, 1794

**XYLOMYIDAE** Verrall, 1901

***XYLOMYA*** Rondani, 1861

*Xylomya czekanovskii* Pleske, 1925

= *interrupta* auct. nec. (Pleske, 1926)

= *maculata* auct. nec. (Meigen, 1804)

superfamily Xylophagoidea Fallén, 1810

**XYLOPHAGIDAE** Fallén, 1810

***XYLOPHAGUS*** Meigen, 1803

= ***Erinna*** Meigen, 1800 suppr.

*Xylophagus ater* Meigen, 1804 see Notes

= *compeditus* Wiedemann, 1820

*Xylophagus cinctus* (De Geer, 1776)

*Xylophagus inermis* Krivosheina & Krivosheina, 2000 see Notes

= *matsumurae* misid.

*Xylophagus junki* (Szilády, 1932)

*Xylophagus kowarzi* (Pleske, 1925) see Notes

= *ater* auct. nec. Meigen, 1804

superfamily Rhagionoidea Latreille, 1802

**RHAGIONIDAE** Latreille, 1802

RHAGIONINAE Latreille, 1802

***RHAGIO*** Fabricius, 1775

*Rhagio annulatus* (De Geer, 1776)

*Rhagio lineola* Fabricius, 1794

*Rhagio maculatus* (De Geer, 1776)

*Rhagio notatus* (Meigen, 1820)

*Rhagio scolopaceus* (Linnaeus, 1758)

*Rhagio tringarius* (Linnaeus, 1758)

CHRYSOPILINAE Bezzi, 1903

***CHRYSOPILUS*** Macquart, 1826

*Chrysopilus auratus* (Fabricius, 1805)

?= *cristatus* (Fabricius, 1775) nom. dubium

*Chrysopilus luteolus* (Fallén, 1814)

*Chrysopilus nubecula* (Fallén, 1814)

? *Chrysopilus suomianus* (Szilády, 1934) see Notes

SPANIINAE Rondani, 1856

***OMPHALOPHORA*** Becker, 1900

*Omphalophora oculata* Becker, 1900

= *lapponica* Frey, 1911

***PTIOLINA*** Zetterstedt, 1842

*Ptiolina nigra* Zetterstedt, 1842

*Ptiolina nigrina* Wahlgren, 1854 see Notes

*Ptiolina nitida* Wahlgren, 1854

*Ptiolina obscura* (Fallén, 1814)

***SPANIA*** Meigen, 1830

*Spania nigra* Meigen, 1830

***SYMPHOROMYIA*** Frauenfeld, 1867

**sg. *Paraphoromyia*** Becker, 1921

*Symphoromyia crassicornis* (Panzer, 1806)

**ATHERICIDAE** Nowicki, 1873

***ATHERIX*** Meigen, 1803

*Atherix ibis* (Fabricius, 1798)

superfamily Tabanoidea Latreille, 1802

**TABANIDAE** Latreille, 1802

CHRYSOPSINAE Lutz, 1905

tribe Chrysopsini Lutz, 1905

***CHRYSOPS*** Meigen, 1803

**sg. *Chrysops*** Meigen, 1803

*Chrysops caecutiens* (Linnaeus, 1758)

*Chrysops divaricatus* Loew, 1858

*Chrysops nigripes* Zetterstedt, 1838

= *lapponicus* Loew, 1858

*Chrysops relictus* Meigen, 1820

= *melanopleurus* Wahlberg, 1848

*Chrysops rufipes* Meigen, 1820

*Chrysops sepulcralis* (Fabricius, 1794)

*Chrysops viduatus* (Fabricius, 1794)

= *pictus* Meigen, 1820

TABANINAE Latreille, 1802

tribe Haematopotini Enderlein, 1922

***HAEMATOPOTA*** Meigen, 1803

*Haematopota crassicornis* Wahlberg, 1848

*Haematopota italica* Meigen, 1804

*Haematopota pluvialis* (Linnaeus, 1758)

= *italica* misid.

? *Haematopota subcylindrica* Pandellé, 1883 see Notes

***HEPTATOMA*** Meigen, 1803

*Heptatoma pellucens* (Fabricius, 1776)

tribe Tabanini Latreille, 1802

***ATYLOTUS*** Osten Sacken, 1876

*Atylotus fulvus* (Meigen, 1820)

*Atylotus plebeius* (Fallén, 1817)

*Atylotus rusticus* (Linnaeus, 1767)

*Atylotus sublunaticornis* (Zetterstedt, 1842)

***HYBOMITRA*** Enderlein, 1922

*Hybomitra arpadi* (Szilády, 1923)

*Hybomitra astuta* (Osten Sacken, 1876) see Notes

= *polaris* (Frey, 1915)

*Hybomitra auripila* (Meigen, 1820) see Notes

= *aterrima* (Meigen, 1820)

*Hybomitra bimaculata* (Macquart, 1826)

= *tropica* misid.

?= *solstitialis* (Meigen, 1820) see Notes

*Hybomitra borealis* (Fabricius, 1781)

= *lapponicus* (Wahlberg, 1848)

*Hybomitra ciureai* (Séguy, 1937)

= *schineri* Lyneborg, 1959

*Hybomitra distinguenda* (Verrall, 1909)

*Hybomitra kaurii* Chvála & Lyneborg, 1970

= *borealis* misid.

*Hybomitra lundbecki* Lyneborg, 1959

= *fulvicornis* misid.

*Hybomitra lurida* (Fallén, 1817)

*Hybomitra montana* (Meigen, 1820)

*Hybomitra muehlfeldi* (Brauer, 1880)

= *flaviceps* (Zetterstedt, 1842)

*Hybomitra nigricornis* (Zetterstedt, 1842)

*Hybomitra nitidifrons* (Szilády, 1914)

= *confinis* misid.

*Hybomitra sexfasciata* (Hine, 1923)

= *borealis anderi* Kauri, 1951

*Hybomitra tarandina* (Linnaeus, 1758)

*Hybomitra tropica* (Linnaeus, 1758)

***TABANUS*** Linnaeus, 1758

*Tabanus autumnalis* Linnaeus, 1761

*Tabanus bovinus* Linnaeus, 1758

*Tabanus bromius* Linnaeus, 1758

*Tabanus cordiger* Meigen, 1820

*Tabanus maculicornis* Zetterstedt, 1842

*Tabanus sudeticus* Zeller, 1842

## Checklist part 2: Asilomorpha

suborder Brachycera Macquart, 1834

clade Orthorrapha Brauer, 1863

superfamily Asiloidea Latreille, 1802

**ASILIDAE** Latreille, 1802

ASILINAE Latreille, 1802

***ASILUS*** Linnaeus, 1758

*Asilus crabroniformis* Linnaeus, 1758

***DIDYSMACHUS*** Lehr, 1996

*Didysmachus picipes* (Meigen, 1820)

***DYSMACHUS*** Loew, 1860

*Dysmachus trigonus* (Meigen, 1804)

***MACHIMUS*** Loew, 1849

*Machimus setibarbis* Loew, 1849

***NEOITAMUS*** Osten Sacken, 1878

*Neoitamus cothurnatus* (Meigen, 1820)

*Neoitamus cyanurus* (Loew, 1849)

*Neoitamus socius* (Loew, 1871)

***NEOMOCHTHERUS*** Osten Sacken, 1878

*Neomochtherus pallipes* (Meigen, 1820)

***PAMPONERUS*** Loew, 1849

*Pamponerus germanicus* (Linnaeus, 1758)

***PHILONICUS*** Loew, 1849

*Philonicus albiceps* (Meigen, 1820)

***RHADIURGUS*** Loew, 1849

*Rhadiurgus variabilis* (Zetterstedt, 1838)

***TOLMERUS*** Loew, 1849

*Tolmerus atricapillus* (Fallén, 1814)

*Tolmerus pyragra* (Zeller, 1840)

LAPHRINAE Macquart, 1838

tribe Andrenosomatini Hull, 1962

***ANDRENOSOMA*** Rondani, 1856

*Andrenosoma albibarbe* (Meigen, 1820)

tribe Laphrini Macquart, 1838

***CHOERADES*** Walker, 1851

*Choerades fuliginosus* (Panzer, 1798)

*Choerades gilvus* (Linnaeus, 1758)

*Choerades igneus* (Meigen, 1820)

*Choerades lapponicus* (Zetterstedt, 1842)

*Choerades marginatus* (Linnaeus, 1758)

***LAPHRIA*** Meigen, 1803

*Laphria flava* (Linnaeus, 1761)

*Laphria gibbosa* (Linnaeus, 1758)

LEPTOGASTRINAE Schiner, 1862

***LEPTOGASTER*** Meigen, 1803

*Leptogaster cylindrica* (De Geer, 1776)

*Leptogaster guttiventris* Zetterstedt, 1842

STENOPOGONINAE Hull, 1962

tribe Dioctriini Hendel, 1936

***DIOCTRIA*** Meigen, 1803

*Dioctria atricapilla* Meigen, 1804

*Dioctria cothurnata* Meigen, 1820

*Dioctria hyalipennis* (Fabricius, 1794)

*Dioctria oelandica* (Linnaeus, 1758)

*Dioctria rufipes* (De Geer, 1776)

tribe Stegopogonini Hull, 1962

***CYRTOPOGON*** Loew, 1847

*Cyrtopogon flavimanus* (Meigen, 1820)

*Cyrtopogon lapponicus* (Zetterstedt, 1838)

*Cyrtopogon lateralis* (Fallén, 1814)

*Cyrtopogon luteicornis* (Zetterstedt, 1842)

= luteicornis var. pollinosus Frey, 1911

*Cyrtopogon pulchripes* Loew, 1871

tribe Stichopogonini Hardy, 1930

***LASIOPOGON*** Loew, 1847

*Lasiopogon cinctus* (Fabricius, 1781)

*Lasiopogon septentrionalis* Lehr, 1984

**BOMBYLIIDAE** Latreille, 1802

PHTHIRIINAE Becker, 1913

tribe Phthiriini Becker, 1913

***PHTHIRIA*** Meigen, 1803

*Phthiria pulicaria* (Mikan, 1796)

BOMBYLIINAE Latreille, 1802

tribe Bombyliini Latreille, 1802

***BOMBYLIUS*** Linnaeus, 1758

**sg. *Bombylius*** Linnaeus, 1758

*Bombylius discolor* Mikan, 1796

*Bombylius major* Linnaeus, 1758

*Bombylius minor* Linnaeus, 1758

= *allibarbis* Zetterstedt, 1842

= *albibarbis* emend.

***SYSTOECHUS*** Loew, 1855

*Systoechus ctenopterus* (Mikan, 1796)

= *sulphureus* (Mikan, 1796)

*Systoechus gradatus* (Wiedemann, 1820)

ANTHRACINAE Latreille, 1804

tribe Anthracini Latreille, 1804

***ANTHRAX*** Scopoli, 1763

*Anthrax anthrax* (Schrank, 1781)

*Anthrax trifasciatus* Meigen, 1804

= *leucogaster* Wiedemann, 1820

*Anthrax varius* Fabricius, 1794

tribe Exoprosopini Becker, 1913

***EXOPROSOPA*** Macquart, 1840

*Exoprosopa capucina* (Fabricius, 1781)

***MICOMITRA*** Bowden, 1964

*Micomitra stupida* (Rossi, 1790)

tribe Villini Hull, 1973

***HEMIPENTHES*** Loew, 1869

*Hemipenthes maura* (Linnaeus, 1758)

*Hemipenthes morio* (Linnaeus, 1758)

***THYRIDANTHRAX*** Osten Sacken, 1886

*Thyridanthrax fenestratus* (Fallén, 1814)

***VILLA*** Lioy, 1864

*Villa cingulata* (Meigen, 1804)

? *Villa halteralis* (Kowarz, 1883) see Notes

*Villa hottentotta* (Linnaeus, 1758)

*Villa modesta* (Meigen, 1820)

*Villa occulta* (Wiedemann, 1820)

**MYTHICOMYIIDAE** Melander, 1902

GLABELLULINAE Cockerell, 1914

***GLABELLULA*** Bezzi, 1902

*Glabellula arctica* (Zetterstedt, 1838)

**SCENOPINIDAE** Burmeister, 1835

***SCENOPINUS*** Latreille, 1802

*Scenopinus fenestralis* (Linnaeus, 1758)

*Scenopinus niger* (De Geer, 1776)

*Scenopinus* sp. A see Notes

= *vitripennis* misid.

**THEREVIDAE** Newman, 1834

THEREVINAE Newman, 1834

***ACROSATHE*** Irwin & Lyneborg, 1981

*Acrosathe annulata* (Fabricius, 1805)

***DIALINEURA*** Rondani, 1856

*Dialineura anilis* (Linnaeus, 1761)

***DICHOGLENA*** Irwin & Lyneborg, 1981

*Dichoglena nigripennis* (Ruthe, 1831)

***PANDIVIRILIA*** Irwin & Lyneborg, 1981

*Pandivirilia eximia* (Meigen, 1820)

***PSILOCEPHALA*** Zetterstedt, 1838

*Psilocephala imberbis* (Fallén, 1814)

***SPIRIVERPA*** Irwin & Lyneborg, 1981

*Spiriverpa lunulata* (Zetterstedt, 1838)

= *clausa* (Frey, 1911)

***THEREVA*** Latreille, 1796

*Thereva cinifera* Meigen, 1830

= *subfasciata* Schummel, 1830

*Thereva fuscinervis* Zetterstedt, 1838

*Thereva handlirschi* Kröber, 1912

= *praestans* Collin, 1948

*Thereva inornata* Verrall, 1909

*Thereva lanata* Zetterstedt, 1838

*Thereva microcephala* Loew, 1847

*Thereva nobilitata* (Fabricius, 1775)

*Thereva plebeja* (Linnaeus, 1758)

*Thereva strigata* (Fabricius, 1794)

*Thereva unica* (Harris, 1780)

= *bipunctata* Meigen, 1820

*Thereva valida* Loew, 1847

= *circumscripta* auct. nec. Loew, 1847

## Checklist part 3: families of uncertain position (*incertae sedis*)

suborder Brachycera Macquart, 1834

clade Orthorrapha Brauer, 1863

? superfamily Nemestrinoidea Griffith & Pidgeon, 1832

**ACROCERIDAE** Leach, 1815

***ACROCERA*** Meigen, 1803

= ***Paracrocera*** Mik, 1886

**sg. *Acrocera*** Meigen, 1803

*Acrocera orbiculus* (Fabricius, 1787)

= *globulus* (Panzer, 1804)

= *borealis* Zetterstedt, 1838

***OGCODES*** Latreille, 1796

**sg. *Ogcodes*** Latreille, 1796

*Ogcodes borealis* Cole, 1919 see Notes

*Ogcodes gibbosus* (Linnaeus, 1758)

*Ogcodes nigripes* (Zetterstedt, 1838) see Notes

*Ogcodes pallipes* Latreille in Olivier, 1812

## Excluded species

*Anastoechus nitidulus* (Fabricius, 1794) labeling mistake

*Beris geniculata* Curtis, 1830 misidentified

*Cliorismia ardea* (Fabricius, 1794) not found within present borders

*Cliorismia rustica* (Panzer, 1804) not found within present borders

*Choerades fimbriata* (Meigen, 1820) mistake

*Choerades ursulus* (Loew, 1851) misidentified see Notes

*Chrysopilus splendidus* (Meigen, 1820) mistake

*Cyrtopogon maculipennis* (Macquart, 1834) labeling mistake

*Epitriptus arthriticus* (Zeller, 1840) mistake

*Machimus gonatistes* (Zeller, 1840) not found within present borders

*Odontomyia hydroleon* (Linnaeus, 1758) not found within present borders

*Pandivirilia nigroanalis* (Kröber, 1928) misidentified

*Phthiria canescens* Loew, 1846 not found within present borders

*Tabanus miki* Brauer, 1880 misidentified

*Tolmerus cingulatus* (Fabricius, 1781) mistake

*Villa fasciata* (Meigen, 1804) not found within present borders

= *circumdata* (Meigen, 1820)

= *venusta* (Meigen, 1820)

*Villa longicornis* Lyneborg, 1965 not found within present borders

*Villa panisca* (Rossi, 1790) not found within present borders

= *circumdata* auct. nec. (Meigen, 1820)

*Xylophagus matsumurae* Miyatake, 1965 misidentified

## Notes

***Choerades ursulus* (Loew, 1851)** is a poorly known taxon. It was synonymized with *Choerades fuliginosus* by Lehr (1991) but later considered valid by Bosák and Hradský (2001). Kahanpää and Winqvist (2005) accepted it as a Finnish species but upon re-examination we consider it most likely that the single Finnish specimen previously identified as *Choerades ursulus* is a dark male of *Choerades fuliginosus*.

***Chrysopilus suomianus* (Szilády, 1934)**. The type locality of this species is Enontekiö, Finland (Szilády 1934). Unfortunately the type material seems lost and the name is probably best treated as a *nomen dubium*. Based on Szilády’s original description it may be a dark form of *Chrysopilus nubecula*.

***Hybomitra astuta* (Osten Sacken, 1876)**. Kahanpää and Winqvist (2005) could not locate any material in Finnish collections. Several new records of this species have since been made and its presence in Finland is now confirmed.

***Hybomitra auripila* (Meigen, 1820)**. The synonymy of *Hybomitra auripila* (Meigen, 1820) with *Hybomitra aterrima* (Meigen, 1820) was established by Schacht (1994) and is accepted here. Schiner (1862) already mentioned *Hybomitra aterrima* as synonym to *Hybomitra auripila*. Since we consider him to be the first revisor, the name *Hybomitra auripila* is valid under the current Code.

***Hybomitra solstitialis* (Meigen, 1820)** has long been known to be a problematic taxon. It is separated from *Hybomitra bimaculata* (Macquart, 1826) based on color characters alone. The examined Finnish material includes a range of intermediates between typical *Hybomitra bimaculata* and *Hybomitra solstitialis* forms. It seems likely that the two names are synonymous, but types should be consulted before synonymy is formally published.

***Haematopota subcylindrica* Pandellé, 1883**. First recorded from Finland by Vuorimies (1984). Unfortunately the specimens listed in his paper could not be found and their identification remains somewhat doubtful.

***Ogcodes borealis* Cole, 1919**. A single Finnish specimen collected in the mid-19^th^ century is the sole Palearctic record of this species. Originally identified and published by Hackman (1970), the record was later confirmed by Kahanpää and Winqvist (2005). *Ogcodes borealis* Cole *sensu*Schlinger (1960) may be a species complex.

***Ogcodes nigripes* (Zetterstedt, 1838)** is probably a senior synonym of *Ogcodes zonatus* Erichson, 1840.

***Ptiolina nigrina* Wahlgren, 1854** may be a synonym of *Ptiolina nigra* Zetterstedt, 1842.

***Scenopinus* sp. A** is an apparently undescribed species near *Scenopinus fenestralis* with black femora. It occurs widely in Finland in association with bird nests.

***Villa halteralis* (Kowarz, 1883)**. See Kahanpää and Winqvist (2005) for a discussion of the single supposed Finnish record of this species.

***Xylophagus ater* Meigen, 1804**. This name has widely been used for two species. Old Finnish checklists (Frey et al. 1941, Hackman 1980) followed the model also used in the world checklist Woodley (2011c) and used this name for the species also known as *Xylophagus kowarzi* (Pleske, 1925). On the British Isles the name *Xylophagus ater* is used as a senior name for *Xylophagus compeditus* Wiedemann in Meigen, 1820. According to Alexander and Clements (1991) and Chandler (1998a, b) the British usage is correct and it is followed here. Thus, *Xylophagus ater* is the common species with females easily identified by the three stripes of dusting on the mesonotum.

***Xylophagus inermis* Krivosheina & Krivosheina, 2000** was described as a subspecies of *Xylophagus matsumurae* Miyatake, 1965 = *maculatus* Matsumura, 1916 (preoccupied by *Xylophagus maculatus* Meigen, 1804) (Krivosheina and Krivosheina 2000). It was raised to a full species status in the recent world catalogue (Woodley 2011c). All collected Finnish specimens formerly identified as *Xylophagus matsumurae* were examined and they belong to *Xylophagus inermis*.

***Zabrachia tenella* (Jaennicke, 1866)**. First recorded from Finland by Krivosheina and Rozkošný (1990). We have examined the Finnish *Zabrachia* material and confirmed the presence of both *Zabrachia tenella* and *Zabrachia minutissima* in the country.
